# EM23, a natural sesquiterpene lactone, targets thioredoxin reductase to activate JNK and cell death pathways in human cervical cancer cells

**DOI:** 10.18632/oncotarget.6828

**Published:** 2016-01-07

**Authors:** Fang-Yuan Shao, Sheng Wang, Hong-Yu Li, Wen-Bo Chen, Guo-Cai Wang, Dong-Lei Ma, Nai Sum Wong, Hao Xiao, Qiu-Ying Liu, Guang-Xiong Zhou, Yao-Lan Li, Man-Mei Li, Yi-Fei Wang, Zhong Liu

**Affiliations:** ^1^ Guangzhou Jinan Biomedicine Research and Development Center, Guangdong Provincial Key Laboratory of Bioengineering Medicine, National Engineering Research Center of Genetic Medicine, Jinan University, Guangzhou, China; ^2^ College of Pharmacy, Jinan University, Guangzhou, China; ^3^ Faculty of Health Sciences, University of Macau, Macau SAR, China; ^4^ Department of Biochemistry, Li Ka Shing Faculty of Medicine, The University of Hong Kong, Hong Kong; ^5^ University of The Chinese Academy of Sciences, Beijing, China

**Keywords:** thioredoxin, thioredoxin reductase, sesquiterpene lactone, ROS, apoptosis

## Abstract

Sesquiterpene lactones (SLs) are the active constituents of a variety of medicinal plants and found to have potential anticancer activities. However, the intracellular molecular targets of SLs and the underlying molecular mechanisms have not been well elucidated. In this study, we observed that EM23, a natural SL, exhibited anti-cancer activity in human cervical cancer cell lines by inducing apoptosis as indicated by caspase 3 activation, XIAP downregulation and mitochondrial dysfunction. Mechanistic studies indicated that EM23-induced apoptosis was mediated by reactive oxygen species (ROS) and the knockdown of thioredoxin (Trx) or thioredoxin reductase (TrxR) resulted in a reduction in apoptosis. EM23 attenuated TrxR activity by alkylation of C-terminal redox-active site Sec498 of TrxR and inhibited the expression levels of Trx/TrxR to facilitate ROS accumulation. Furthermore, inhibition of Trx/TrxR system resulted in the dissociation of ASK1 from Trx and the downstream activation of JNK. Pretreatment with ASK1/JNK inhibitors partially rescued cells from EM23-induced apoptosis. Additionally, EM23 inhibited Akt/mTOR pathway and induced autophagy, which was observed to be proapoptotic and mediated by ROS. Together, these results reveal a potential molecular mechanism for the apoptotic induction observed with SL compound EM23, and emphasize its putative role as a therapeutic agent for human cervical cancer.

## INTRODUCTION

Thioredoxin reductase (TrxR) is a ubiquitously expressed oxidoreductase crucial for maintaining cellular redox homeostasis by counteracting the effects of reactive oxygen species (ROS) and regulating redox-related signaling cascades [1]. TrxR is the only enzyme known to catalyze the reduction of thioredoxin (Trx), which participates in wide variety of cellular processes including the oxidative stress response, gene regulation via the NF-κB, p53, and Ref-1 transcription factors, and the competitive inhibition of apoptosis-regulating signal kinase 1 (ASK1)-induced apoptosis [2].

Elevated Trx/TrxR activity was found to be involved in the development and progression of cancer [3, 4]. Notably, over expression of Trx/TrxR can promote cancer cell proliferation and has been associated with tumor angiogenesis, invasion, and metastasis [5]. Furthermore, the Trx/TrxR system contributes to tumor cell resistance to oxidative stress-induced apoptosis [6], which is an important mechanism of various anticancer agents. In addition, knock down of TrxR expression nearly abolished the capacity of lung cancer cells to form tumors in a xenograft model system [7]; over expression of the alternative splicing variant of TrxR induced apoptosis in HeLa cells [8]. Overall, these studies substantiate the notion that mammalian TrxR may be a promising drug target for cancer therapy.

The C-terminal active site of mammalian TrxR has a conserved sequence, -Gly-Cys-Sec-Gly-COOH, which contains an active selenocysteine. During catalysis, electrons are transferred from NADPH to the N-terminal active site of TrxR, where they are delivered to the C-terminal redox-active sites (Cys^497^ and Sec^498^) [9]. Then, TrxR can subsequently reduce various substrates, including the disulfide active site of Trx. The inhibitory effects of many active compounds and drugs targeting mammalian TrxR are mainly based on their interactions with the Sec^498^ residue. Prominent examples include gold- and platinum-based compounds [10], arsenic trioxide [11], curcumin [12], and natural flavonoids [13].

Sesquiterpene lactones (SLs) are the active constituents of a variety of medicinal plants. In recent years, the anticancer activity of SLs has attracted a great deal of interest and extensive research has been carried out to illuminate their anticancer properties [14]. SLs have been reported to inhibit proliferation, migration, invasion and induce apoptosis in various tumor cells, such as lung cancer [15], breast cancer [16], melanoma [17], leukaemia [18], and colorectal cancer [19]. Main molecular events involved in these SLs-specific activities were investigated to be inhibition of NF-κB activation, suppression of anti-apoptotic proteins, and blockage of PI3K/Akt and JAK2/STAT3 signaling [19–22]. However, the intracellular molecular targets of SLs have not been well elucidated and continued work is needed to identify the mechanisms underlying the potential function of SLs in cancer therapy.

In this study, we demonstrate the anticancer activity of EM23, a natural SL family member isolated from *Elephantopus mollis*, in the CaSki and SiHa human cervical cancer cell lines. Mechanistically, we demonstrate that EM23 specifically targets the mammalian TrxR by alkylating the C-terminal redox-active site Sec^498^ of this enzyme. EM23-induced ROS accumulation and ASK1/JNK signal activation are critical for the induction of apoptosis. Furthermore, EM23 treatment also facilitates autophagy, which contributes to the post-treatment apoptosis observed in CaSki and SiHa cells.

## RESULTS

### Growth inhibitory effect of EM23 on human cancer cell lines

**Figure 1:**
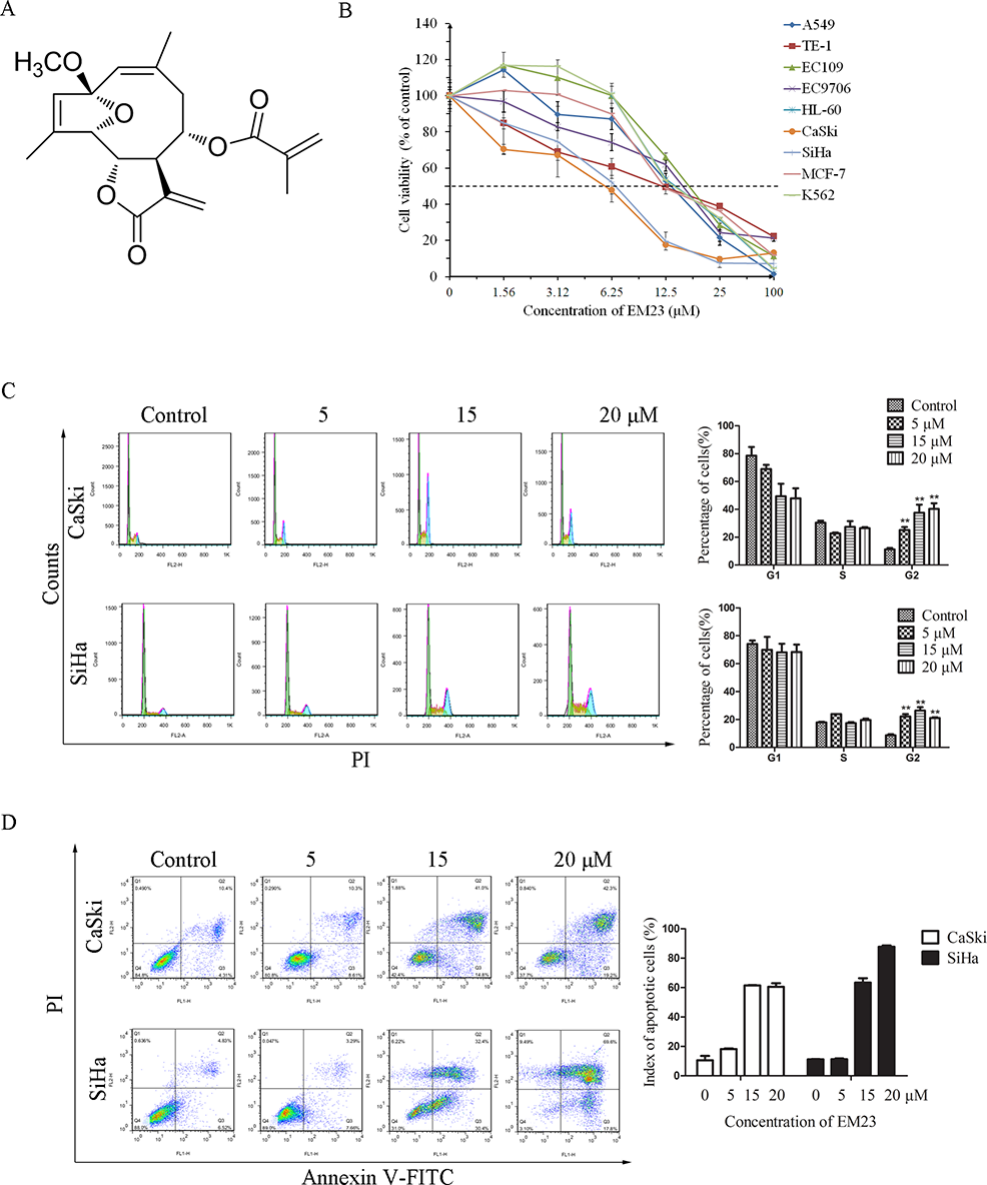
EM23 induced cell apoptosis and cell cycle arrest (**A**) Chemical structure of EM23. (**B**) Effects of EM23 on the growth of the following human cancer cell lines: lung cancer cell line A549; breast cancer cell line MCF-7; esophageal cancer cell lines TE-1, EC109, and EC9706; cervical cancer cell lines CaSki and SiHa; and leukemia cell lines HL-60 and K562. Cells were treated with various concentrations of EM23 for 24 h, and cell viability was measured by MTT assay. (**C**) Effects of EM23 on cell cycle distribution. CaSki and SiHa cells were treated with 0, 5, 15, and 20 μM EM23 for 24 h, and cell cycle distribution was measured by flow cytometry after PI staining. (**D**) Induction of apoptosis by EM23 in CaSki and SiHa cells. Cells were treated as above and index of apoptotic cells were analyzed by flow cytometry after Annexin-V-FITC/PI staining. All data are presented as the mean ± SD of three independent experiments. *P < 0.5 and **P < 0.01.

The chemical structure of EM23 is shown in Figure 1A. To determine the effect of EM23 on the cellular growth inhibition, several human cancer cell lines were treated with varying concentrations of EM23 for 24 h, including lung cancer cell line A549; human breast cancer cell line MCF-7; human esophageal cancer cell lines TE-1, EC109, and EC9706; human cervical cancer cell lines CaSki and SiHa; and human leukemia cell lines HL-60 and K562. Cell viability was assessed by MTT assay. As shown in Figure 1B, EM23 exhibited the most significant growth inhibitory activities on cervical CaSki and SiHa cell lines. The IC_50_ values of EM23 for CaSki and SiHa cells were 5.8 and 6.6 μM, respectively. In contrast, significantly lesser loss of cell viability due to treatment with EM23 was observed on human cervical epithelial Ect1/E6E7 cell line (Supplementary Figure S1). The IC_50_ value of EM23 for Ect1/E6E7 cells was 85.5 μM, which was approximately 15-fold and 13-fold higher than that of for CaSki and SiHa cells, respectively. The molecular mechanism(s) underlying the effects of EM23 on the human cervical cancer cell lines was next investigated.

### EM23 induces cell cycle arrest and apoptosis in CaSki and SiHa cells

To examine the mechanism underlying the inhibitory effects of EM23 on cell growth, the cell cycle phase distributions of EM23-treated CaSki and SiHa cells were analyzed by flow cytometry. Notably, an increase in the G2/M population was observed in cells following treatment with EM23, as treatment with 20 μM EM23 increased the G2/M population of CaSki and SiHa cells by 29% and 13%, respectively, as compared to control groups (Figure 1C).

To investigate EM23-induced apoptotic cell death, both cell lines were treated with increasing concentrations of EM23 for 24 h and stained with Annexin-V-FITC/PI. The resulting apoptotic ratios were analyzed by flow cytometry. As shown in Figure 1D, EM23 elicited apoptosis in a dose-dependent manner in both cell lines, as treatment with 20 μM EM23 resulted in 60% and 87% cell death in CaSki and SiHa cells, respectively. The TUNEL assay for observation of apoptotic DNA cleavage also confirmed EM23-induced apoptosis (Supplementary Figure S2).

### EM23 induces caspase 3 activation and mitochondrial dysfunction

**Figure 2:**
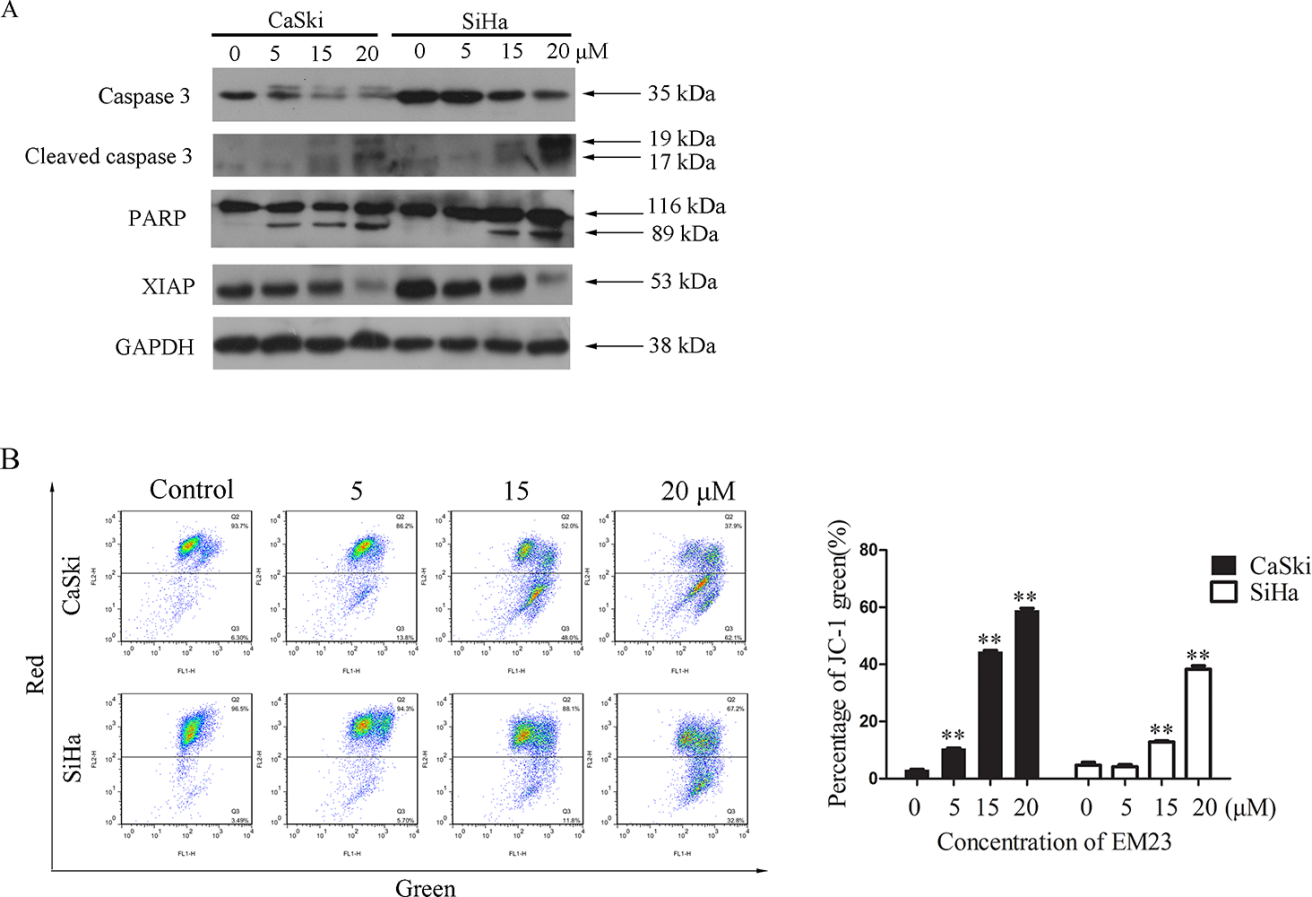
Effects of EM23 on caspase 3 and mitochondrial membrane potential (MMP) (**A**) Western blotting analysis of the expression levels of the following: caspases 3, cleaved caspases 3, PARP and XIAP. (**B**) Effect of EM23 on MMP in CaSki and SiHa cells with the treatment of 0, 5, 15, and 20 μM EM23 for 24 h. The MMP were analyzed by flow cytometry after JC-1 staining. Cells with MMP loss were gated. All data are representatives of three independent experiments or presented as the mean ± SD of three independent experiments. *P < 0.5 and **P < 0.01.

We next analyzed the effect of EM23 on caspase 3 activation, PARP cleavage, and XIAP protein expression. Significantly, western bloting analysis revealed that EM23 induced caspase 3 cleavage, and downregulated the expression level of pro-caspase 3 (Figure 2A). PARP, a DNA-repair enzyme, is a known substrate of caspase 3 [23]; therefore, we examined PARP degradation following EM23 treatment. As shown in Figure 2A, PARP underwent specific proteolytic cleavage as suggested by the generation of the 89 kDa PARP fragment in CaSki cells treated with EM23 at 5, 15 and 20 μM and in SiHa cells treated with EM23 at 15 and 20 μM, respectively. In addition, the expression level of XIAP, the most potent human IAP protein that can inhibit the activity of caspase 3 was observed to decrease with EM23 treatment in a dose-dependent manner.

Mitochondria play a central role in the intrinsic apoptosis pathway. The loss of mitochondrial membrane potential (MMP; Δψ_m_) is regarded as a hallmark of cell apoptosis that occurs prior to caspase activation [24]. As shown in Figure 2B, EM23 treatment significantly disrupted the Δψ_m_ in both cell lines, as demonstrated in a shift from red to green. Notably, the relative percentage of fluorescence green cells increased by 54% and 35% in CaSki and SiHa cells, respectively, following treatment with 20 μM EM23.

### EM23 induced-ROS generation is involved in its anticancer activity

**Figure 3:**
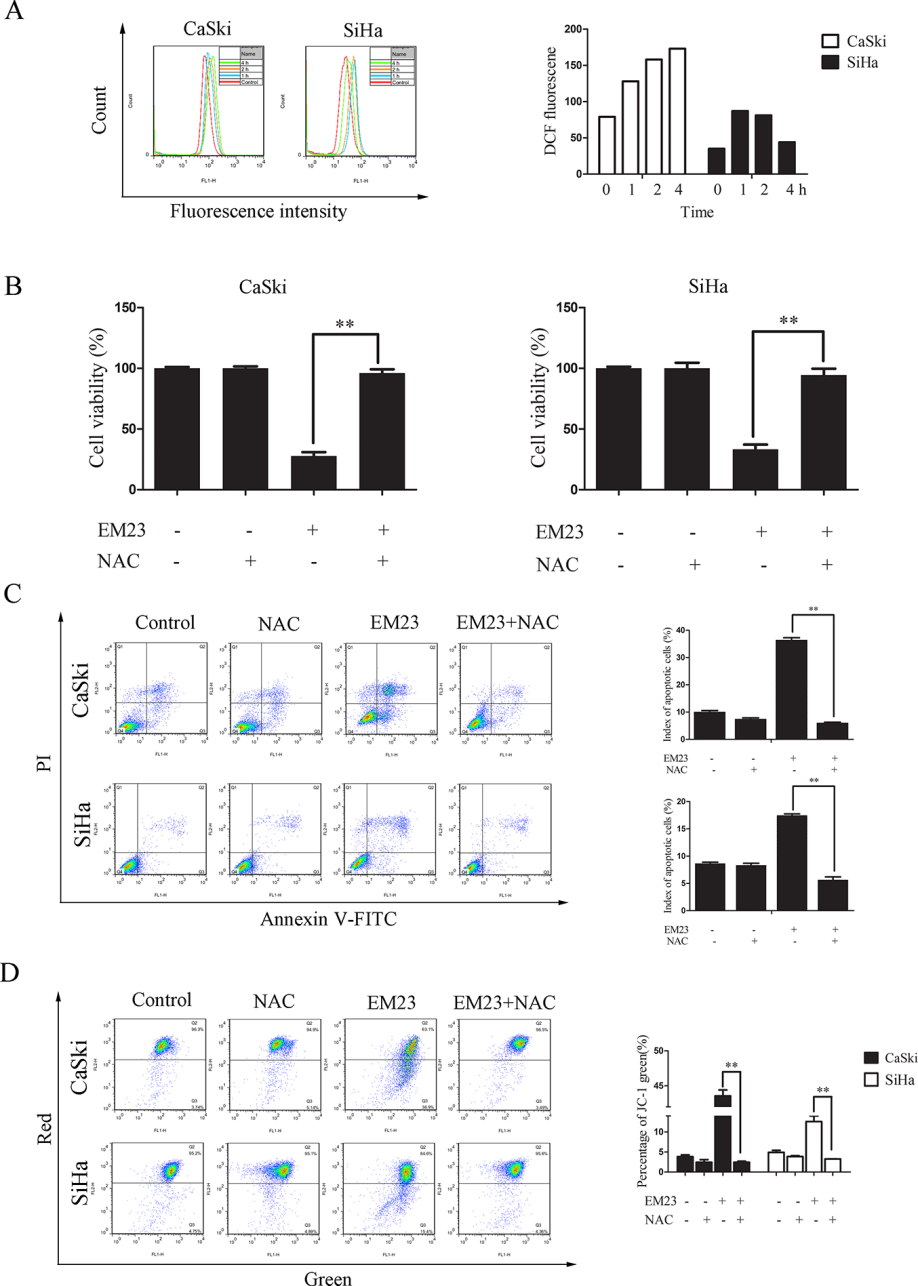
The role of ROS production in EM23-induced cell apoptosis (**A**) EM23-induced ROS production in CaSki and SiHa cells treated with 15 μM EM23 for 1-4 h. Cells were stained with DCFH-DA, and analyzed for fluorescence by flow cytometry at 525 nm. (**B**) EM23-induced growth inhibition in CaSki and SiHa cells was inhibited by NAC pre-treatment. Cells were pretreated with 10 mM NAC, and then treated with 15 μM EM23 for 24 h. Cell viability was then analyzed by MTT assay. (**C**) EM23-induced apoptosis was inhibited by NAC pre-treatment. CaSki and SiHa cells were treated as above and index of apoptotic cells were analyzed by flow cytometry after Annexin-V-FITC/PI staining. (**D**) EM23-induced MMP disruption was inhibited by NAC pre-treatment. Cells were treated as above and index of MMP were analyzed by flow cytometry after JC-1 staining. All values are presented as the mean ± SD of three independent experiments. *P < 0.5 and **P < 0.01.

Mitochondria are the main source of ROS, and excess ROS accumulation elicits oxidative stress that can cause prominent damage to DNA, lipids, and proteins within the cell to subsequently induce apoptosis [25]. To investigate the role of ROS generation on the anticancer activity of EM23, we first examined intracellular ROS production in EM23-treated CaSki and SiHa cells using DCFH-DA. As shown in Figure 3A, EM23 caused a remarkable accumulation of ROS in CaSki and SiHa cells, particularly following the 4 and 2 h treatments, respectively. Notably, pretreatment with NAC, a ROS scavenger, almost completely rescued EM23-induced apoptosis in both cell lines, indicating that ROS accumulation is involved in EM23-induced apoptosis (Figure 3B and 3C). Furthermore, EM23 induced Δψ_m_ disruption was also completely rescued by NAC pre-treatment (Figure 3D).

### EM23 inhibits the activity of TrxR in cell-free systems

**Figure 4:**
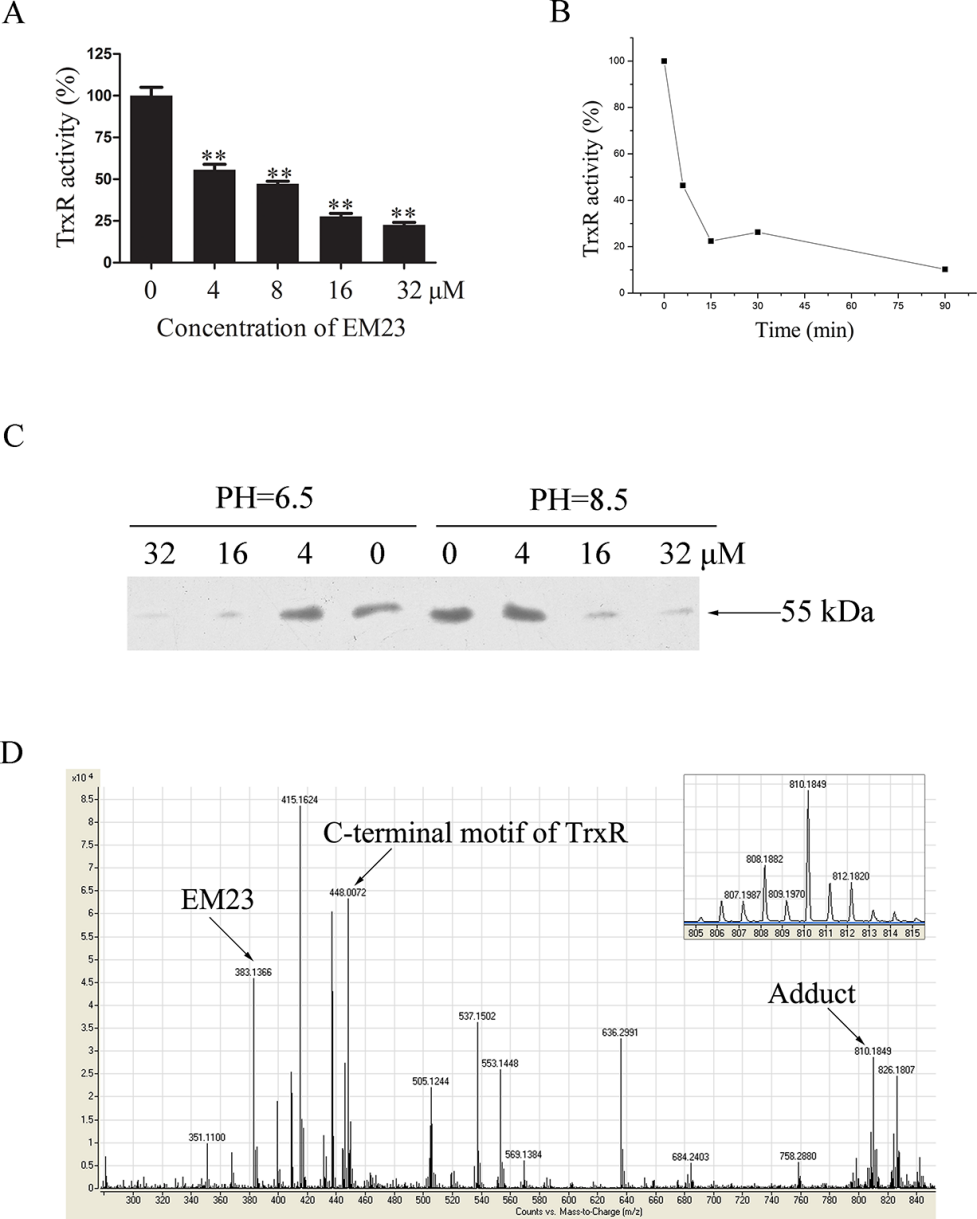
EM23 inhibited TrxR activity by binding to the selenocysteine site (**A**) NADPH-reduced TrxR was incubated with various concentrations of EM23 in cell-free system at room temperature for 5 min. TrxR activity was measured by DTNB reduction assay. (**B**) NADPH-reduced TrxR was incubated with10 μM EM23 for 5, 15, 30 and 90 min. TrxR activity was measured by DTNB reduction assay. (**C**) Different concentrations of EM23 were added to reduced TrxR and incubated at room temperature for 1 h, then subjected to BIAM alkylation assays. The same amounts of DMSO were added to the control groups. (**D**) TOF LC/MS analysis of the adduct formed by TrxR C-terminal peptide Ac-Gly-[Cys-Sec]-Gly-NH_2_ and EM23. The *m/z* signal peaks corresponding to EM23, tetrapeptide of C-terminal motif of TrxR and adduct were indicated by arrows, respectively. Up right, the zoomed in signal peak of *m/z* 810.2. All data are representatives of three independent experiments.

To investigate the effects of EM23 on TrxR activity, NADPH-reduced TrxR was incubated with EM23 in cell-free system. As shown in Figure 4A, TrxR activity was inhibited by EM23 in a dose-dependent manner with an IC_50_ value of 7.8 μM as determined by DTNB assay. In addition, EM23-induced TrxR inactivation was also positively correlated with incubation time (Figure 4B). TrxR activity was almost completely inhibited by EM23 after 90 min of incubation. These results identify EM23 as an effective TrxR inhibitor.

### EM23 attenuates TrxR activity by binding the selenocysteine site

The three-dimensional crystal structure of mammalian TrxR revealed two redox-active sites (Cys^497^ and Sec^498^) located on the flexible C-terminal arm of the enzyme [9]. In its reduced form, TrxR-Cys^497^ and -Sec^498^ were present as –SH and –SeH groups, respectively. It has been reported that both –SH and –SeH groups can be alkylated at high pH (pH 8.5), while only the –SeH group is alkylated at low pH (pH 6.5) [26]. As such, we applied this finding to identify whether EM23 targeted these active sites by performing BIAM alkylating reactions in buffers with different pH values.

As shown in Figure 4C, a weaker band intensity was observed when the reduced form of TrxR was incubated with EM23 prior to BIAM labeling, indicating that TrxR was first alkylated by EM23. Notably, at low pH (pH 6.5), band intensity weakened dramatically with high concentrations of EM23, indicating that C-terminal redox-activesite Sec^498^ can be alkylated by EM23. At a high pH (pH 8.5), the band intensity weakened at the same extent observed in low pH (pH 6.5) buffer, indicating that the TrxR-Sec^498^ redox-active site is the only specific target for EM23 modification.

To further confirm this result, a short TrxR C-terminal peptide Ac-Gly-[Cys-Sec]-Gly-NH_2_ was synthesized and reacted with EM23 to determine whether EM23 could specifically bind the selenol group of TrxR. The representative TOF LC/MS spectrum of this reaction products were shown in Figure 4D. The molecular mass of the *m/z* 383.1 ([M+Na]^+^) and 448.0 ([M+Na]^+^) signal peak exactly matches that of EM23 and the tetrapeptide, respectively. The molecular mass signal identified at *m/z* 810.2 ([M+Na]^+^) showed the distinctive isotopic pattern characteristic of selenium (Figure 4D, up right), indicating that an equivalent amount of EM23 covalently bound to the tetrapeptide. However, no signal at *m/z* 1170.4 ([M+Na]^+^) was detected (data not shown), suggesting that there existed no adduct of the tetrapeptide bearing two EM23 groups in this reaction. This MS result confirmed TrxR-Sec^498^ redox-active site as the specific target for EM23.

### Docking simulation between EM23 and the active site of TrxR

**Figure 5:**
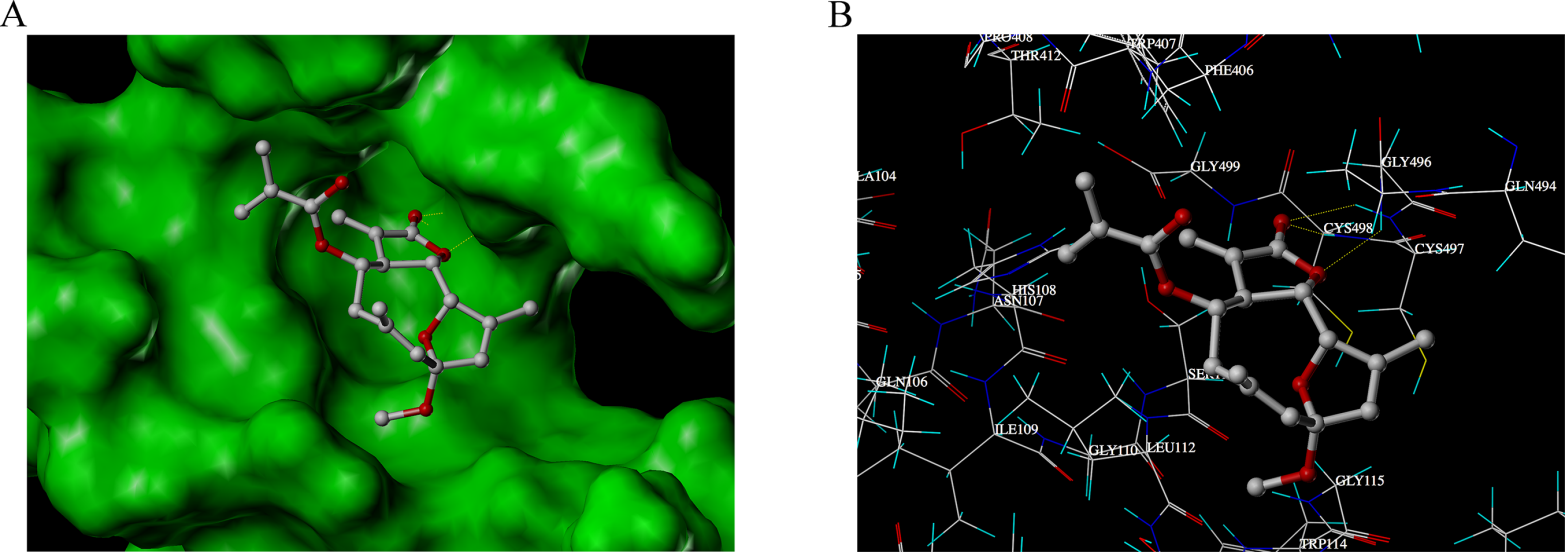
The binding mode research between EM23 and TrxR by docking simulations (**A**) The MOLCAD surface of the binding pocket of docked TrxR-EM23 complexes was displayed in Fast Connolly pattern. (**B**) Binding interactions with selected residues of the active site of TrxR for EM23.

In order to further investigate the binding modes of EM23 with TrxR, molecular docking studies were carried out by Surflex-Dock suite implemented in SYBYL 8.0 software. The results showed that the most energetically favorable binding mode of EM23 at the active site of TrxR comed into a free binding energy of −5.08 kcal/mol. The MOLCAD surface modeling displayed in Fast Connolly pattern demonstrated that EM23 extended into the pocket of active site of TrxR (Figure 5A). The lactonic ring of EM23 was close to the C-terminal Cys^497^/Cys^498^ motif of TrxR (The Sec^498^ has been substituted by a Cys residue in the crystal structure of TrxR [27]). The distances of carbonyl motif at the lactonic ring of EM23 between backbone NH of Cys^497^ and Cys^498^ were 2.01 and 1.95 Å, respectively, which may allow hydrogen bonding and contribute to covalent interactions between EM23 and side chain of Cys^497^ and/or Cys^498^. Additionally, the oxygen atom at the lactonic ring of EM23 forms another hydrogen bond with the backbone NH of Gly^496^.

### EM23 inhibits the expression levels of Trx/TrxR in CaSki and SiHa cells

**Figure 6:**
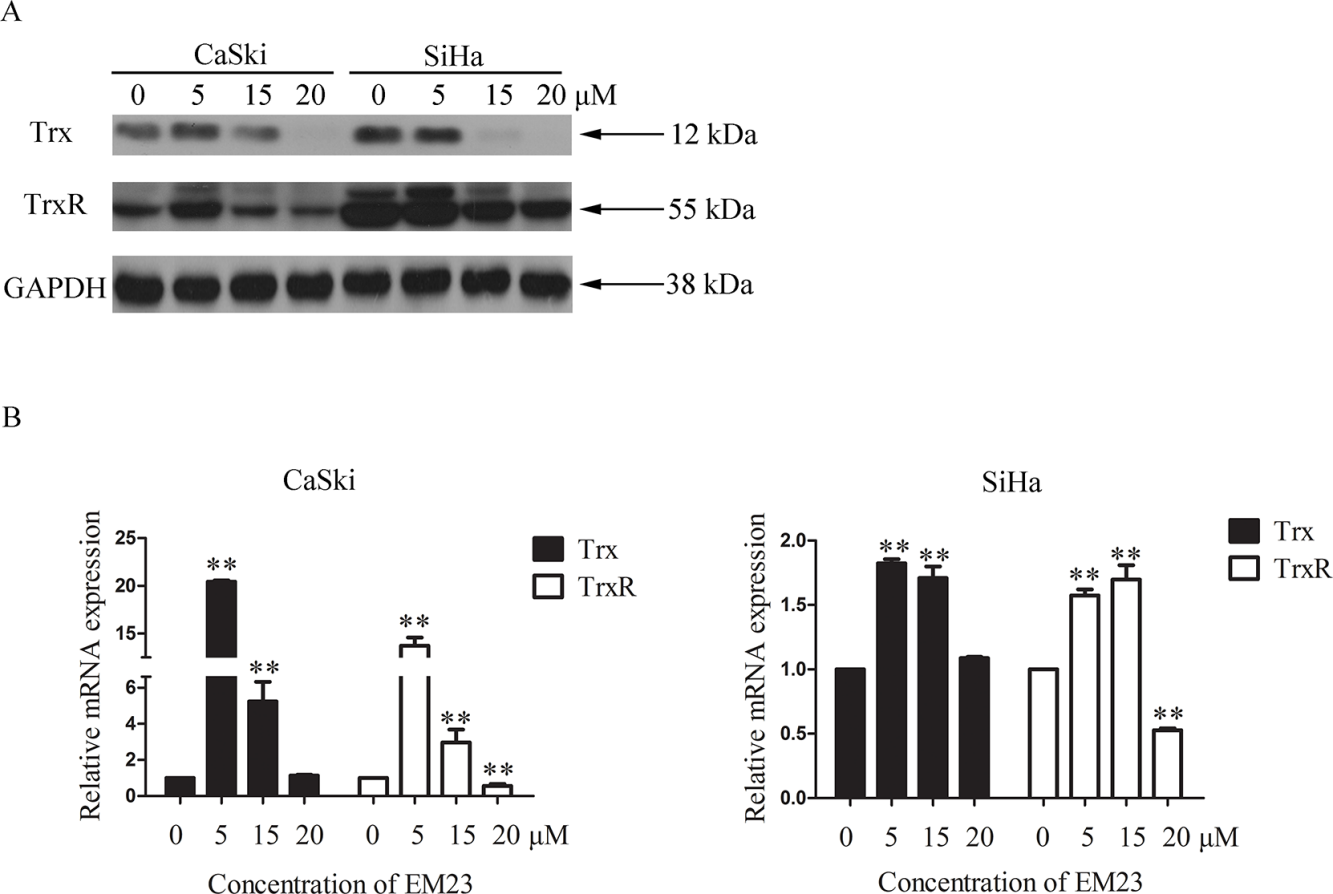
Effects of EM23 on the expression levels of Trx/TrxR system (**A**) CaSki and SiHa cells were treated with 0, 5, 15 and 20 μM EM23 for 24 h, and Trx and TrxR expression levels were analyzed by western blotting. (**B**) The mRNA levels of Trx and TrxR were analyzed by RT-PCR; GAPDH was used as an internal control. Data are presented as the mean ± SD of three independent experiments. *P < 0.5 and **P < 0.01.

The Trx/TrxR system plays a crucial role in both cellular redox homeostasis and cell death regulation, and is often found over-expressed in cancer. We next examined effects of EM23 on Trx and TrxR expression in CaSki and SiHa cells. As shown in Figure 6A, Trx protein expression was diminished in both cell lines, and was dose-dependent in SiHa cells. Interestingly, TrxR expression slightly increased with 5 μM EM23 treatment in the CaSki cell line, but then decreased dose-dependently with the 15 and 20 μM treatments. Furthermore, the mRNA expression levels of Trx and TrxR were assessed by RT-PCR assay. As shown in Figure 6B, 5 μM EM23 treatment resulted in an increase of Trx and TrxR mRNA transcription, whereas 15 and 20 μM downregulated their expression levels in both cell lines.

### Knock down of Trx or TrxR results in a reduction of EM23-induced apoptosis

**Figure 7:**
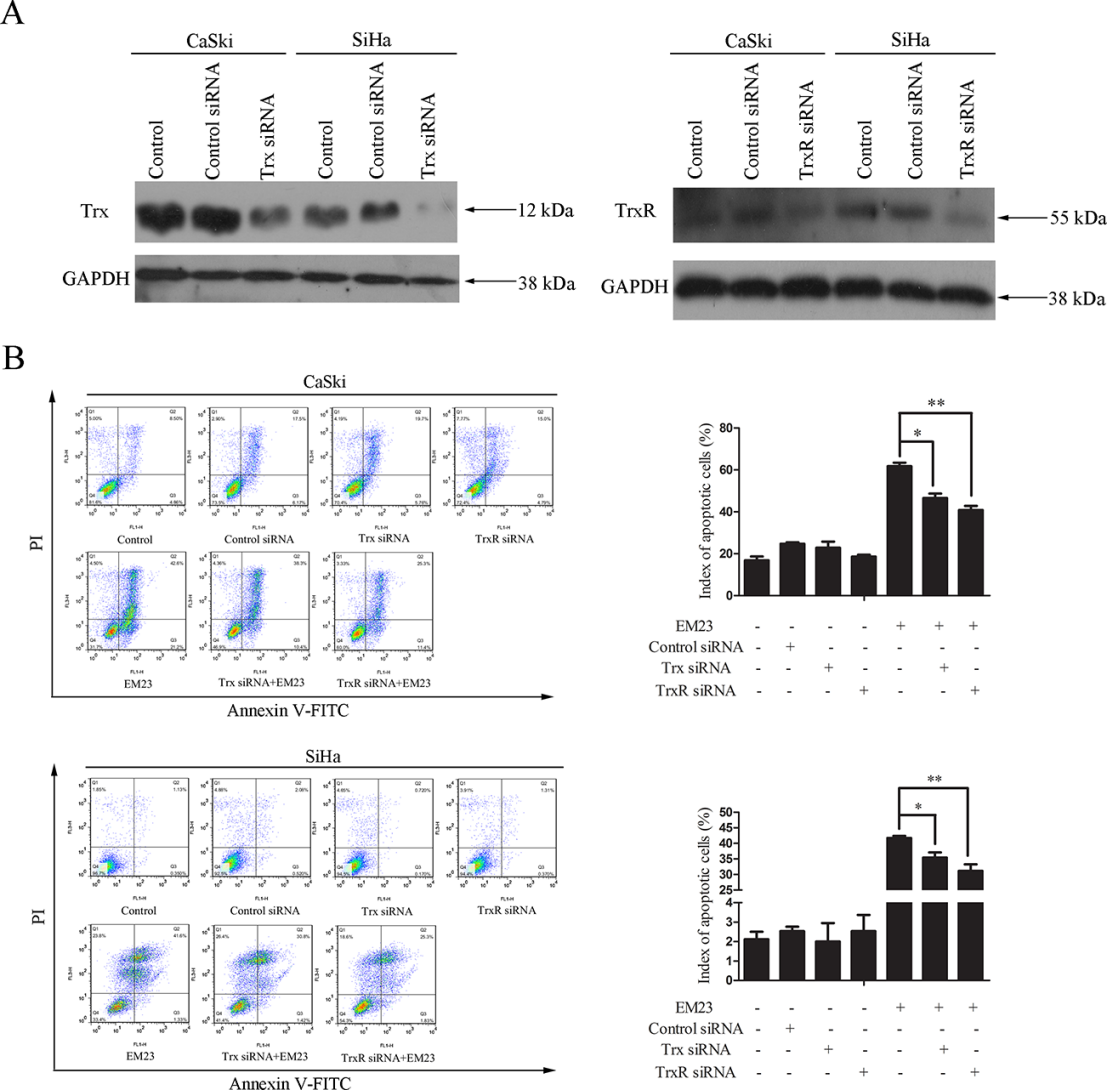
Effect of Trx or TrxR knock down on EM23-induced apoptosis CaSki and SiHa cells were transfected with Trx or TrxR siRNA. (**A**) Protein lysates were prepared 24 h after transfection, and the expression levels of Trx and TrxR were analyzed by western blotting. (**B**) After transfection for 24 h, CaSki and SiHa cells were treated with 15 μM EM23 for another 24 h. Apoptotic cells were then analyzed by flow cytometry after Annexin-V-FITC/PI staining. Data are presented as the mean ± SD of three independent experiments. *P < 0.5 and **P < 0.01.

To directly assess the role of the Trx/TrxR system in EM23-induce apoptosis, we utilized siRNAs that specifically target either Trx or TrxR, in addition to a control siRNA. As shown in Figure 7A, transfection of Trx or TrxR siRNA effectively reduced the respective protein levels in both cell lines. EM23-induced apoptosis in siRNA transfected CaSki and SiHa cells was then assessed by Annexin-V-FITC/PI staining and flow cytometry analysis. Significantly, knock down of Trx or TrxR attenuated EM23-induced apoptosis in both cell lines (Figure 7B). These results suggest that targeting the Trx/TrxR system is essential for EM23-induced apoptosis in CaSki and SiHa cells.

### EM23 induces activation of ASK1/JNK signaling

**Figure 8:**
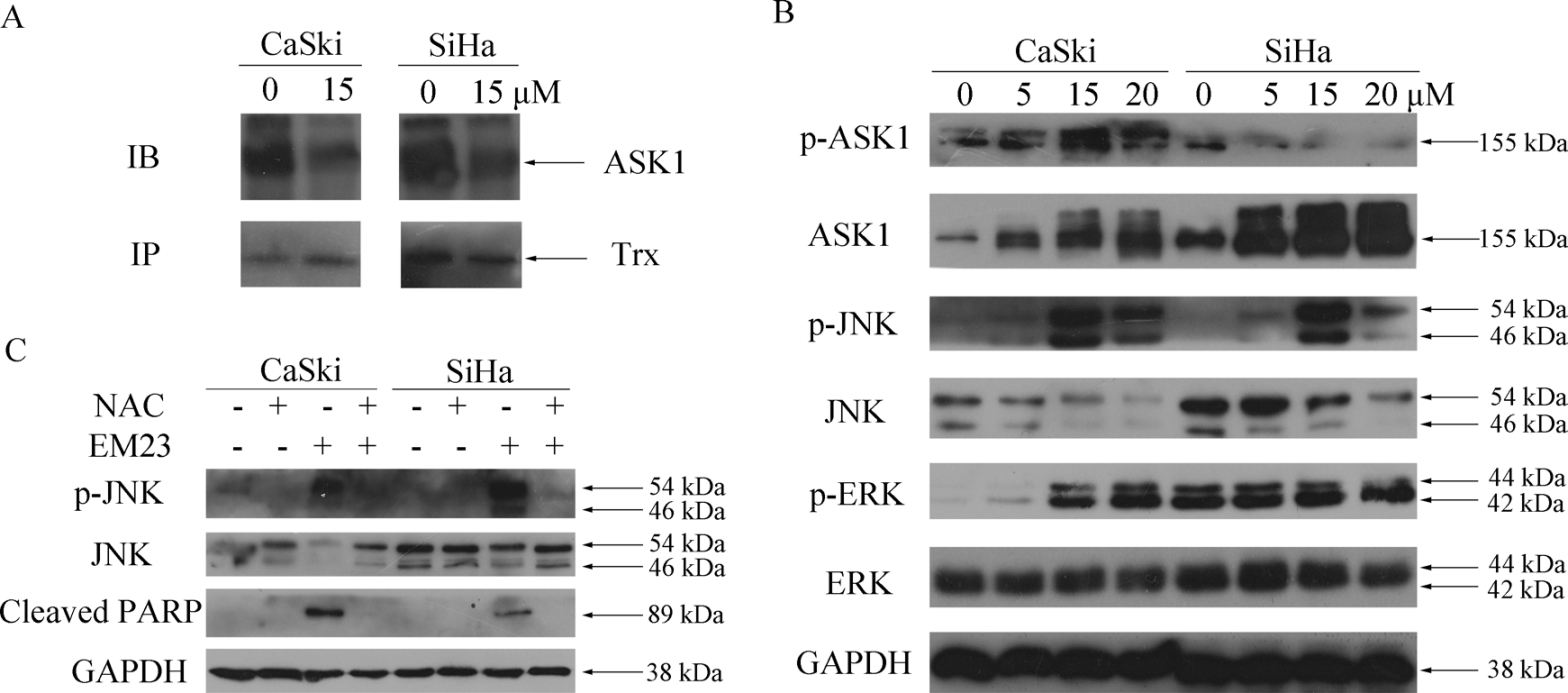
Effects of EM23 on ASK1, JNK and ERK signaling pathways (**A**) CaSki and SiHa cells were treated with 15 μM EM23 for 8 h. ASK1-Trx complex was then precipitated by Protein A beads conjugated with Trx antibodies and analyzed by western blotting with ASK1 and Trx antibodies. (**B**) CaSki and SiHa cells were treated with 0, 5, 15 and 20 μM EM23 for 24 h, the expression levels of ASK1, p-ASK1, JNK, p-JNK, ERK and p-ERK were analyzed by western blotting analysis. (**C**) Effect of ROS scavenger NAC on JNK signaling pathway and PARP. CaSki and SiHa cells were pre-treated with 10 mM NAC followed by 15 μM EM23 for 24 h. Protein lysates were prepared and expression levels of JNK, p-JNK and PARP were analyzed by western blotting analysis. All data are representatives of three independent experiments.

The competitive inhibitory effect of reduced Trx on ASK1 is one of the important mechanisms by which Trx/TrxR system elicits apoptotic resistance in cancer cells [28]. ROS-induced apoptosis can be induced by the oxidization of Trx, and the subsequent release of ASK1, which can then be activated to prompt apoptotic signaling through the p38 and JNK pathway [29]. Here, we investigated Trx-ASK1 complex formation after treatment with 15 μM EM23 by co-immunoprecipitation assay. As expected, we found that ASK1 dissociated from Trx following EM23 treatment in both cell lines (Figure 8A), and consequently gained kinase activity sufficient to elicit downstream signal activation, as demonstrated by the increase in phosphorylated ASK1 and JNK, respectively (Figure 8B). Furthermore, an increase in ASK1 protein expression was observed in both cell lines after EM23 treatment (Figure 8B). Interestingly, we found that extracellular signal-regulated protein kinase 1/2 (ERK1/2) signaling was also activated after EM23 treatment, but only in CaSki cells (Figure 8B).

**Figure 9:**
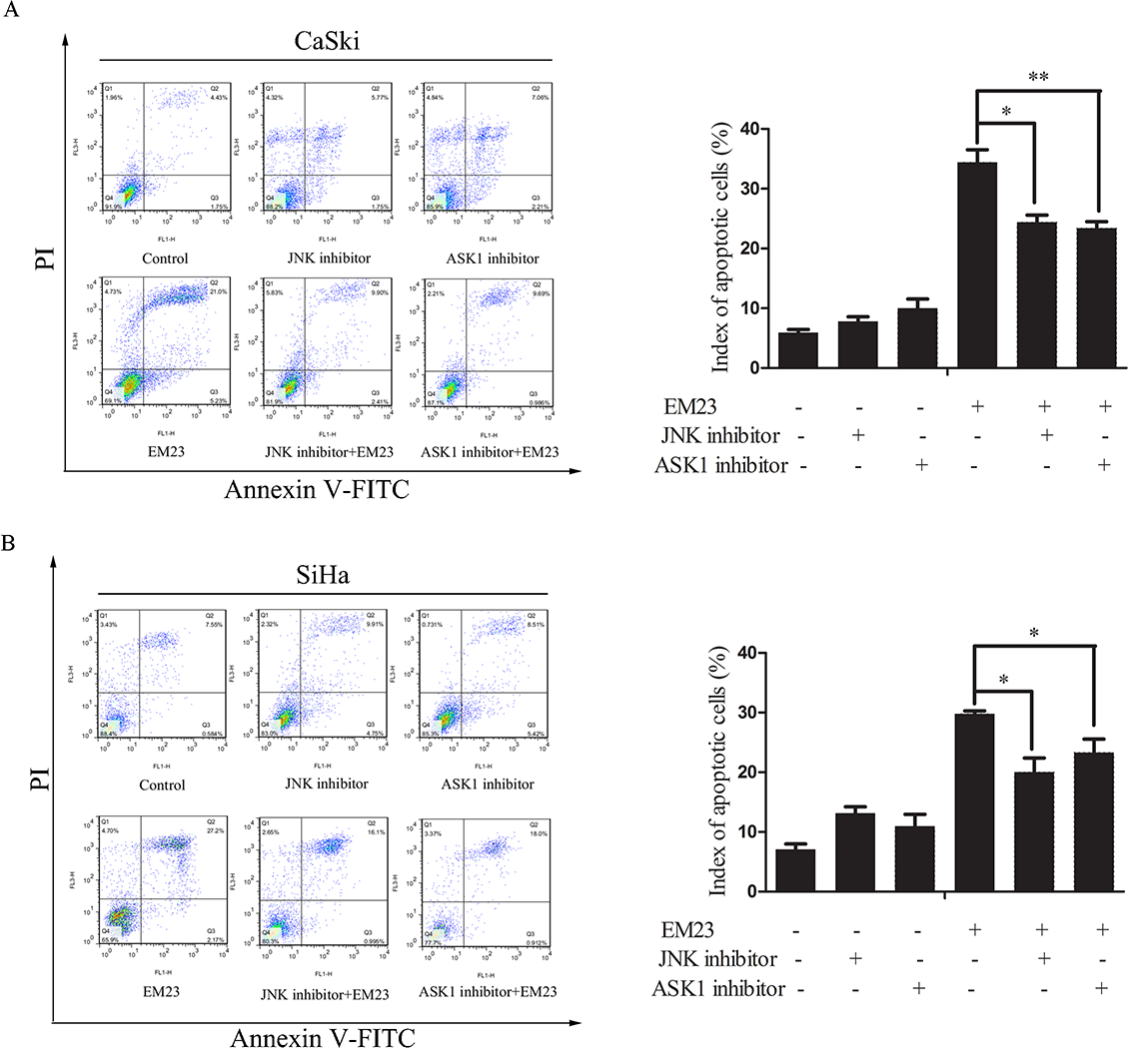
Effects of ASK1 and JNK inhibitor on EM23-induced apoptosis Cells were pre-treated with 0.6 and 0.2 μM ASK1 and JNK inhibitor, respectively, and then incubated with 15 μM EM23 for 24 h. Index of apoptosis in CaSki (**A**) and SiHa (**B**) cells were analyzed by flow cytometry after Annexin-V-FITC/PI staining. Data are presented as the mean ± SD of three independent experiments. *P < 0.5 and **P < 0.01.

To further investigate the role of these kinases in EM23-induced apoptosis, Annexin-V-FITC/PI dual staining flow cytometry analysis was used to analyze the cellular apoptosis induced by the combination of EM23 and ASK1 or JNK inhibitor. As shown in Figure 9, the ASK1 and JNK inhibitors attenuated EM23-induced apoptosis in both cell lines. Notably, the apoptotic ratio declined to about 24% and 23%, respectively, in CaSki cells, and about 20% and 23%, respectively, in SiHa cells, as compared to EM23-only treatment group. These results further support the important role for ASK1/JNK signaling in EM23-induced apoptosis.

JNK signaling pathway is a crucial downstream pathway triggered by the oxidative stress response [30]. As such, we investigated the effect of the anti-oxidant, NAC, on EM23-induced JNK signaling. As shown in Figure 8C, NAC pretreatment blocked EM23-induced JNK activation and PARP cleavage, emphasizing the essential role of ROS in EM23-induced JNK pathway activation and subsequent cell apoptosis.

### EM23 induces autophagy and inhibits Akt/mTOR signaling pathway

**Figure 10:**
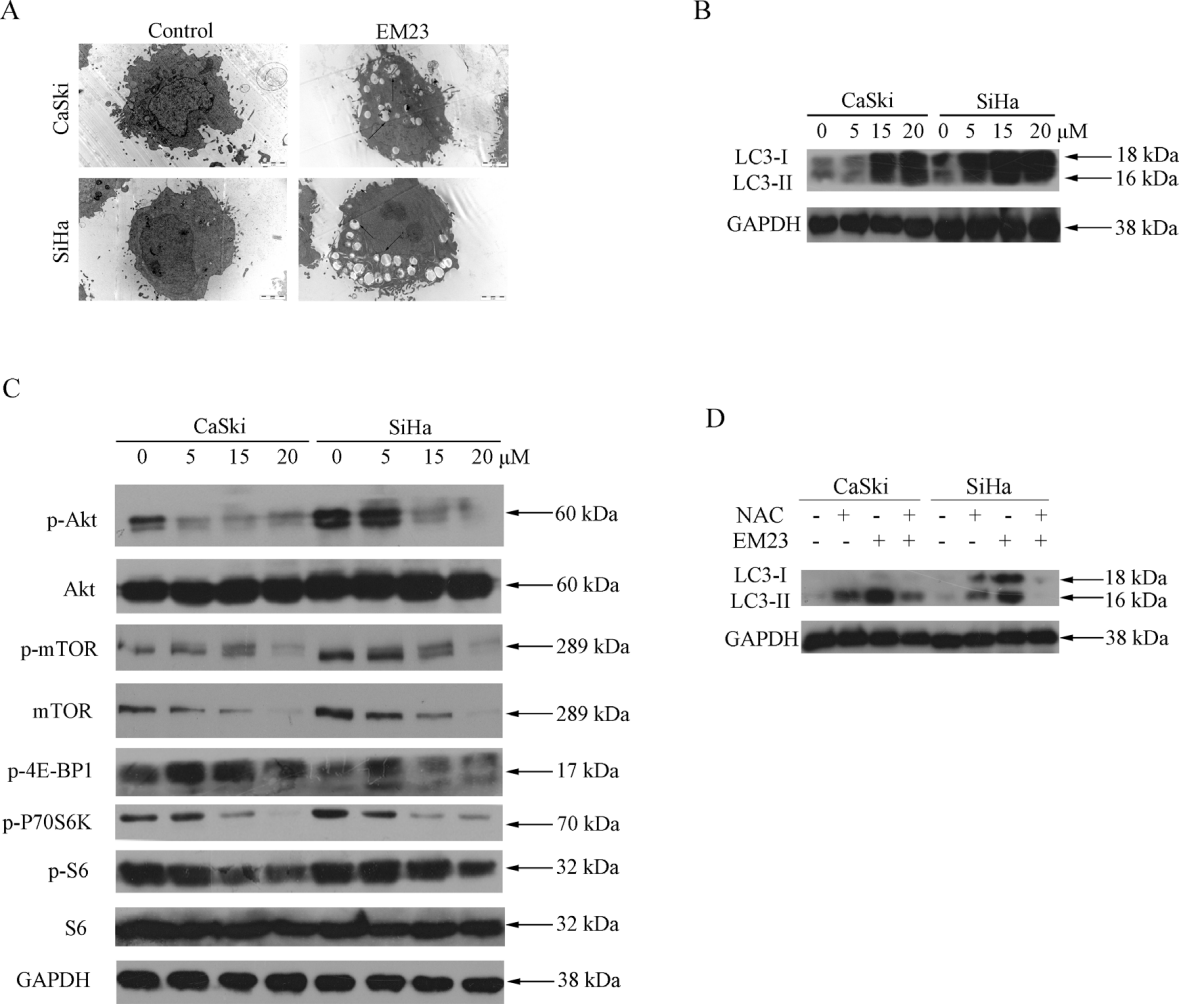
EM23 induced autophagy and inhibited Akt/mTOR signaling pathway in CaSki and SiHa cells (**A**) Microstructure of cells treated with 15 μM EM23 for 24 h; transmission electron microscopy shows multiple vacuoles. (**B**) Western blotting analysis of expression levels of LC3 I/II in CaSki and SiHa cells treated with 0, 5, 15, and 20 μM EM23 for 24 h. (**C**) The expression levels of p-Akt, Akt, p-mTOR, mTOR, p-4E-BP1, p-P70S6K, p-S6, and S6 in CaSki and SiHa cells. Cells were treated with 0, 5, 15, and 20 μM EM23 for 24 h and then subjected to western blotting analysis. (**D**) CaSki and SiHa cells were pretreated with 10 mM NAC and treated with 15 μM EM23 for 24 h, protein lysates were then prepared and the expression levels of LC3 I/II were analyzed by western blotting analysis. All data are representatives of three independent experiments.

Autophagy-related programmed cell death has been observed in the cellular response to several anticancer reagents [31]. In our study, we found that treatment with 15 μM EM23 for 24 h induced autophagy in both CaSki and SiHa cells, as demonstrated by the autophagic vacuoles observed with electron microscopy (Figure 10A). Furthermore, we found that 15 μM EM23 also induced the accumulation of LC3-II (Figure 10B), an autophagy-related, ubiquitin-like modifier, regarded as an autophagosomal marker in mammals cells [32]. These results suggest that EM23 induced autophagy in CaSki and SiHa cells.

The Akt/mTOR-mediated signaling cascade is a key negative regulator of autophagy [33]. As shown in Figure 10C, both Akt and mTOR phosphorylation declined in response to EM23 treatment. The phosphorylation status of 70S6K and 4E-BP1, the substrates of mTOR was then examined [34]. It was observed that the phosphorylation level of 70S6K decreased in both cell lines upon treatment with EM23; however, no significant change in phosphaorylation was observed for that of 4E-BP1 (Figure 10C). It is interesting that EM23 only caused a slight decrease in phosphorylation level of ribosomal protein S6, the downstream target of 70S6K, and had no effect on the expression level of S6 (Figure 10C). Together, these results indicate the involvement of Akt/mTOR signaling pathway in EM23-induced autophagy.

### EM23-induced autophagy is mediated by ROS and promotes apoptosis

Autophagy frequently results from ROS accumulation and mitochondria dysfunction [35]. Notably, we found that pre-treatment with NAC rescued the EM23-induced LC3-II accumulation observed previously (Figure 10D), implying that EM23-induced autophagy was mediated by EM23-induced ROS accumulation.

**Figure 11:**
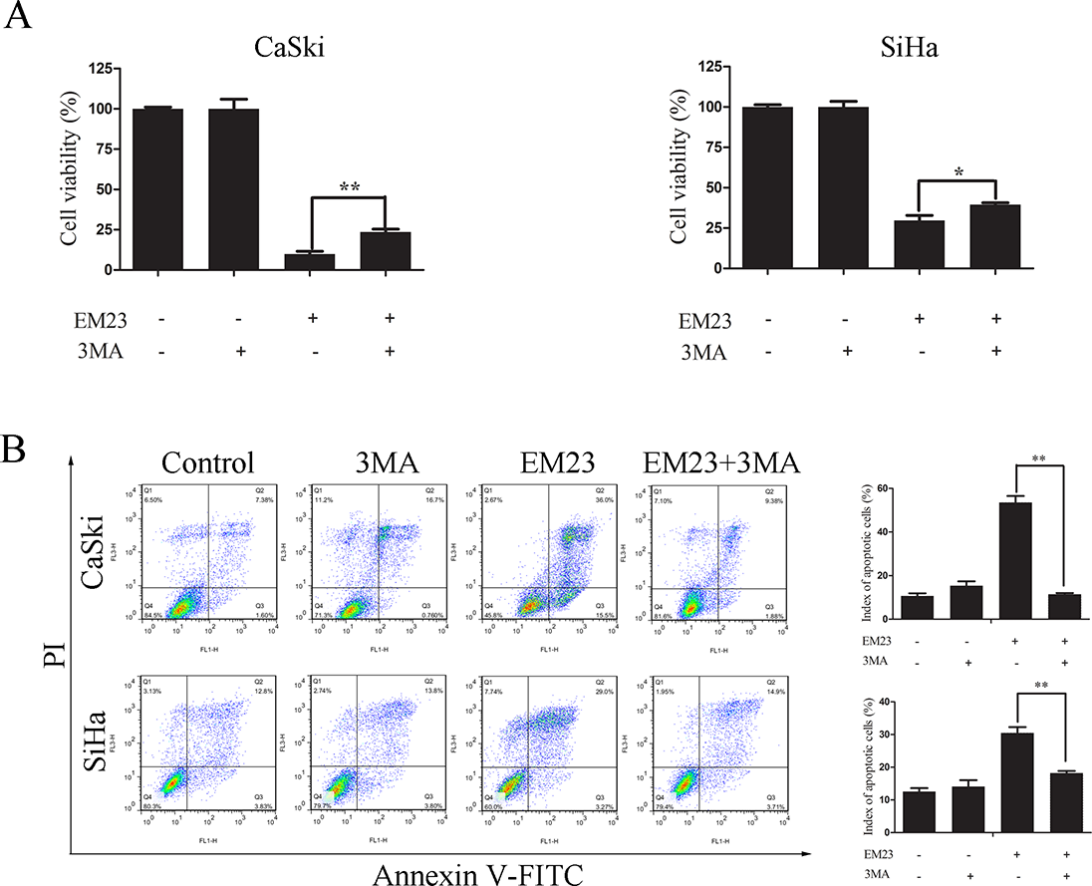
Effects of 3-MA on EM23-induced cell growth inhibition and apoptosis CaSki and SiHa cells were pre-treated with 5 mM 3-MA for 1 h, followed by 15 μM EM23 for 24 h. Cell viability was analyzed by MTT assay (**A**) and the apoptotic index cells were analyzed by flow cytometry after Annexin-V-FITC/PI staining (**B**). All data are presented as the mean ± SD of three independent experiments. *P < 0.5 and **P < 0.01.

To investigate whether EM23-induced autophagy imparted a protective or detrimental effect, cells were pre-treated with 5 mM 3-MA prior to treatment with 15 μM EM23. Cell growth and apoptosis were then determined by MTT assay and Annexin-V-FITC/PI staining, respectively. As shown in Figure 11A, the inhibitory effect of EM23 on cell growth was weakened by 3-MA treatment in both cell lines. These results were further verified by Annexin-V-FITC/PI staining and flow cytometry analysis (Figure 11B). These data indicate that EM23-induced autophagy indeed had a detrimental effect on CaSki and SiHa cells, and resulted in apoptosis.

## DISCUSSION

The Trx/TrxR system, composed of TrxR, Trx, and NADPH, is the major protein disulfide reduction system of the cell [36]. The mammalian TrxR is a Sec-containing oxidoreductase previously identified as a chemotherapeutic target in cancer cells and contains a highly reactive selenocysteine susceptible to electrophilic attack, which makes TrxR a suitable target for anticancer drug development. To date, many synthetic and natural therapeutic compounds that exhibit anticancer properties have been classified as TrxR inhibitors. Prominent examples include platinum drugs [37], gold compounds [38], arsenic trioxide [39], curcumin [12], mercury [40], motexafin gadolinium [41], nitroaromatic compounds [42], and flavonoids [13], all of which have been evaluated either as novel cancer treatments or adjuncts to existing therapies. Our findings demonstrate that EM23 inhibits the reduction activity of TrxR in cell-free systems (Figure 4A), and is also sufficient to induce the downregulation of Trx and TrxR in CaSki and SiHa cells (Figure 6).

Trx and TrxR have important roles in enzymatic reductions and resistance against oxidative stress [43]. Generally, the inhibition of TrxR activity will directly influence the redox functions of Trx, leading to a disruption in cellular redox homeostasis. Disrupted redox status can activate the mitochondria-dependent apoptosis pathway frequently observed in several representative SLs, including helenalin [44], costunolide [45], and parthenolide [46]. Here, we found that EM23 inhibited cell growth, and facilitated apoptosis by promoting caspase and PARP cleavage, as well as disruption of Δψ_m_ (Figure 2). We also found that ROS accumulation was involved in EM23-induced apoptosis in CaSki and SiHa cells, as the ROS scavenger, NAC, almost completely reversed these effects (Figure 3).

It is generally believed that one of the most important molecular mechanisms for SL-induced apoptosis involves the conjugation of their unsaturated carbonyl structures to sulfhydryl groups. TrxR represents a potential target of EM23 because its C-terminal active site contains a highly reactive selenocysteine (Sec^498^) residue essential for the catalytic activity of TrxR. The ability of EM23 to inhibit the TrxR activity identifies it as a molecular target of EM23 (Figure 4). BIAM-labeling assays identified the C-terminal Sec^498^ as the primary site of alkylation by EM23, a result further confirmed by TOF LC-MS analysis of adduct of the C-terminal redox-active motif of TrxR by EM23 and molecular docking stimulation (Figure 5). Notably, the lactonic ring of EM23 was close to the redox-active site of TrxR as demonstrated by the docking stimulation, which may allow the covalent interactions occurring between EM23 and the Cys^497^ and Sec^498^ simultaneously. Nevertheless, it is interesting that both BIAM-labeling assays and TOF LC-MS analysis demonstrated the selective alkylation of EM23 towards only the Sec^498^, but not the neighboring Cys^497^. This may be that the reactivity of the selenocysteine to electrophiles is greater than the cysteine, which was calculated to be approximately 1000 times difference [47]. To our knowledge, our study is the first report that SL compound inhibits TrxR activity by specifically targeting the C-terminal redox-active site Sec^498^.

TrxR inactivation can lead to an imbalance of cellular redox state and regulation of Trx/TrxR system-related cell signal cascades. ASK1, a member of the MAPK kinase kinase family, is an important signaling molecule regulated by Trx/TrxR system. Under normal conditions, inactivated ASK1 is bound to reduced Trx; however, upon oxidative stress, ASK1 activates when Trx redox state shifts to the oxidized form [48]. Indeed, our results demonstrate that ASK1 is released from Trx following EM23 treatment (Figure 8A), and subsequently activates, as demonstrated by the increase in phosphorylation at Ser83 of ASK1. JNK, one of the signal mediators downstream of ASK1, is classified as stress-activated kinase and is activated by a variety of stressors, including oxidative stress and chemotherapeutic drugs [30]. The ROS-ASK1-JNK signaling axis has important roles in oxidative stress-induced cell death and mostly required for oxidative stress-induced apoptosis. JNK has been found to regulate both pro-apoptotic and anti-apoptotic proteins, such as c-Jun, 14-3-3, and Mcl-1 [49], that play key roles in mitochondria-dependent apoptosis. In addition, pretreatment with ASK1 and JNK-specific inhibitors, or expression of dominant negative ASK1 and JNK mutants, hindered cell apoptosis induced by various types of stress [50–52], which is in agreement with our observations (Figure 9), suggesting that EM23-induced apoptosis is dependent on the ASK1/JNK pathway. However, pro-apoptotic JNK activation could also be regulated by other MAP3Ks, in addition to ASK1, such as MEKK1 and MLK3. As such, further studies are needed to determine whether ASK1 plays a critical role in EM23-induced JNK activation.

Sustained exposure to oxidative stress is potentially very harmful to cells. Among several intracellular signaling pathways elicited by oxidative stress, the MAPK cascade plays pivotal roles in regulating oxidative stress-induced cell death [30]. In our study, ROS scavenging, which is upstream signal of MAPK, resulted in a reduction of JNK activation and PARP cleavage (Figure 8C), highlighting the important roles of ROS in EM23-induced activation of JNK signal.

Many cancer cells have high levels of expression of Trx and TrxR; therefore, it is not surprising that knock down of TrxR in a mouse lung cancer cell line nearly abolished their capacity to form xenograft tumors [7]. This seems contradictory to our founding. Interestingly, a reduction of TrxR activity was observed in HeLa cells following both siRNA-mediated knock down or treatment with a therapeutic inhibitors; however, only the therapeutic agents, but not the siRNA, resulted in increased oxidative stress [53]. This indicates that residual TrxR1 activity remaining upon TrxR1 knock down could still be enough to counteract ROS. In addition, some compounds can also convert TrxR to a ROS-generating enzyme and induce cell apoptosis, in direct contrast to its usual role [42, 54, 55]. Furthermore, another report found that the TrxR system was required for caspase 3 activation and apoptosis [56], whereas we found that EM23-induced apoptosis was partially dependent on a normal level of TrxR/Trx. Nevertheless, more evidence is needed to illuminate the precise mechanism of TrxR/Trx-dependent EM23-induced apoptosis, including the effect of TrxR or Trx knockdown on EM23-induced ROS accumulation, caspase 3 activation, and the MAPK pathway activation.

The phosphoinositide 3-phosphate kinase (PI3K)/Akt/mTOR signaling pathway and the Ras/Raf1/ERK1/2 pathway are known to regulate autophagy in the cellular response to nutrient starvation [31]. The PI3K/mTOR and ERK1/2 pathways negatively and positively regulate autophagy, respectively. Accumulating evidence has shown that autophagy is activated in response to various therapeutic agent treatments, including suberoylanilide hydroxamic acid [57], vitamin K2 [58], Lapatinib [59], and ursolic acid [60]. Furthermore, curcumin treatment induced autophagic cell death in human malignant glioma cells by inhibiting the mTOR/p70S6K and activating the ERK1/2 pathways simultaneously [33], whereas triterpenoid B-group soyasaponins worked by inhibiting AKT signaling and enhancing ERK activity [61]. Thus, Akt/mTOR inhibition and ERK activation are probably common mechanisms of autophagy induced by anticancer agents. In addition, recent studies have shown that LC3 is processed by a specific protein activation/conjugation system to form autophagosomal membranes during autophagy [62]. In this study, we found the reduction of Akt/mTOR signaling and the enhanced conversion of LC3-I to LC3-II occurred in a dose-dependent manner (Figure 10B and 10C). It is interesting that a significant decrease was observed in the phosphorylation level of P7S6K with EM23 treatment, whereas there was no obvious change in that of 4E-BP1 in spite of the decrease of mTOR phosphorylation (Figure 10C). It can be explained that the selectivity and sensitivity of mTOR for its substrates may be dependent on the integrity and configuration of mTOR and a change in the phosphorylation status of one mTOR substrate does not necessarily correlate with changes in others [34]. Yip *et al.* have also reported that rapamycin strongly declined P7S6K phosphorylation, whereas its effect on 4E-BP1 was more variable [63]. In addition, it should be noted that EM23 caused a slighter decrease in phosphorylation level of S6 than that of 70S6K. This may be due to the other kinases such as RSK that involve in regulating the phosphorylation of S6 [64]. Although autophagy can be stimulated in many tumor cells under therapeutic agent-induced stress, the exact role of autophagy in cancer cell death is still not clear. Notably, many anticancer drugs, including bortezomib, resveratrol, sorafenib, histone deacetylase inhibitors, chloroquine, sulforaphane, and mTOR inhibitors, similarly engender autophagic cell death [65]. Currently, induction of autophagic cell death has been regarded as a potential method for cancer therapy. Consistent with these findings, we found that EM23-induced autophagy had an apoptosis-promoting effect in both cell lines (Figure 11).

While the precise mechanism of autophagy remains unclear, mitochondrial-originated ROS was thought to play a central role in the induction of autophagy, and could be significantly blocked by NAC, a ROS scavenger. In this study, we found that LC3-II accumulation was blocked by NAC pre-treatment (Figure 10D). It is worthy noted that inhibition of ROS by NAC is able to decrease not only ROS level but also mTOR activity [66]. To a certain degree, the pre-treatment of NAC might contribute to EM-23-induced autophagy and the following apoptosis.

Interestingly, recent investigations have shown that ginseng metabolite [67], isoorientin [35], polygonatum cyrtonema [68]-induced autophagy results from ROS accumulation and subsequent activation of the JNK signaling. In this studies, we found that EM23-induced accumulation of ROS occurred upstream of JNK activation (Figure 8C), implying a causal role of JNK signaling in EM23-induced autophagy. However, more evidence is needed to solidify this mechanism.

**Figure 12:**
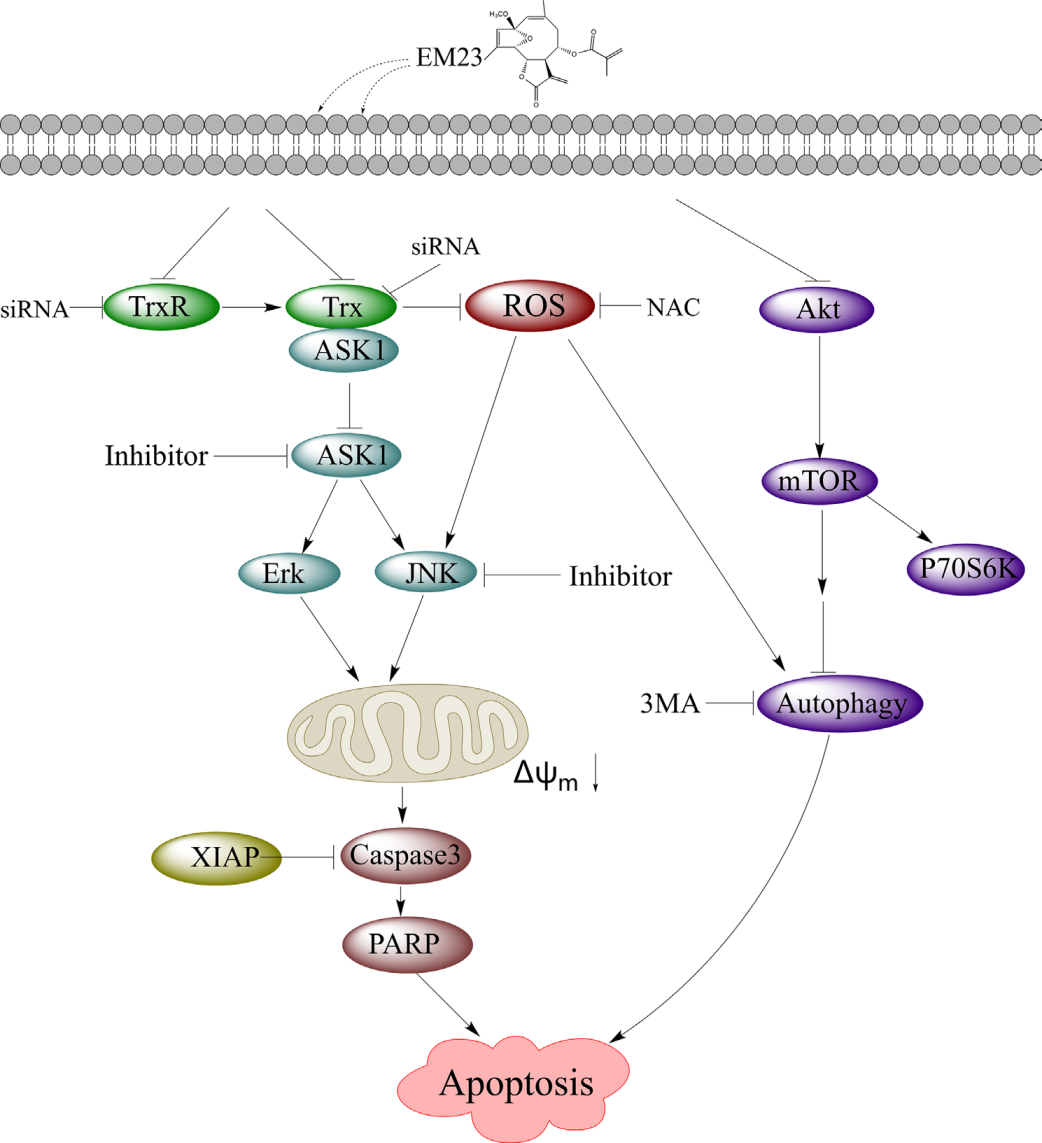
EM23-mediated intracellular signaling elicits apoptosis in human cervical cancer cells

In summary, our study demonstrated that EM23 was an efficient anticancer agent *in vitro*, and was sufficient to induce apoptosis in two human cervical cancer cell lines, CaSki and SiHa (Figure 12). EM23 attenuated TrxR activity by binding to the selenocysteine site of TrxR and inhibited the expression levels of Trx/TrxR to facilitate ROS accumulation, which played a central role in EM23-induced apoptosis. In addition, inhibition of Trx/TrxR system resulted in the dissociation of ASK1 from complex with Trx and activation of downstream JNK signaling pathway, which contributed to EM23-induced apoptosis. ROS also facilitated the activation of JNK signaling pathway. Furthermore, EM23 inhibited Akt/mTOR ribosomal protein-signaling pathway and induced autophagy, which was proapoptotic and mediated by ROS. This report represents the first comprehensive analysis of mammalian TrxR and cellular signaling events in response to the treatment of EM23 in human cervical cancer cells, provides a possible molecular mechanism for the anticarcinogenic effects of SLs and emphasizes the potential application of EM23 as a therapeutic agent for human cervical cancer.

## MATERIALS AND METHODS

### Cell culture and reagents

The cells of the following were purchased from the Cell Bank of the Chinese Academy of Sciences (Shanghai, China): lung cancer cell line A549; human breast cancer cell line MCF-7; human esophageal cancer cell lines TE-1, EC109, and EC9706; human cervical cancer cell lines CaSki and SiHa; and human leukemia cell lines HL-60 and K562. Both HL-60 and K562 cell lines were cultured in RPMI 1640 medium (Life Technologies, USA) and the other cell lines were cultured in DMEM (Gibco; Carlesbad, CA, USA). All the medium were supplemented with 10% fetal bovine serum (FBS, Gibco), 100 U/mL penicillin, and 100 μg/mL streptomycin. The cells were grown at 37°C in a humidified 5% CO_2_ atmosphere.

TrxR was purified from porcine brains, as described by Cheung *et al.* [69]. EM23 was isolated from *Elephantopus mollis* as described previously [70]. Ac-Gly-[Cys-Sec]-Gly-NH_2_ was synthesized by Shanghai Tauto Biotech Co, Ltd. (Shanghai, China) as described by Pratesi *et al.* [71]. Triphosphopyridine nucleotide reduced tetrasodium salt (NADPH), *N*-acetyl-l-cysteine (NAC), Dimethyl sulfoxide (DMSO), 5,5′,6,6′-tetrachloro-1,1′,3,3′-tetraethyl-imidacarbocyanine iodide (JC-1), 2′,7′-dichlorofluoresceindiacetate (DCFH-DA), 3-methyladenine (3-MA) autophagy inhibitor, 3-(4,5-dimetrylthiazol-2-yl)-2,5-diphenyltetrazolium bromide (MTT), 5,5′-dithio-bis (2-nitrobenzoid acid) (DTNB), and glutaraldehyde were purchased from Sigma (St. Louis, MO, USA). JNK inhibitor II (SP600125) and ASK1 inhibitor (ERK kinase kinase 5 inhibitor) were purchased from Merck Millipore (Bellerica, MA, USA). Biotin-conjugated iodoacetamide (BIAM), propidium iodide (PI), dithiothreitol (DTT), and RNase were purchased from Beyotime (Shanghai, China).

Antibodies against ASK1, p-ASK1 (Ser83), GAPDH, caspase 3, cleaved caspase 3, X-linked inhibitor of apoptosis (XIAP), PARP, JNK, p-JNK1/2 (Thr183/Tyr185), ERK, p-ERK (Thr202/Tyr204), p-4E-BP1, p-P70S6K, LC3, mTOR, p-mTOR (Ser2448), S6, and p-S6 (Ser235/236) were purchased from Cell Signaling Technology (CST; Beverly, MA, USA). Trx, TrxR, and horseradish peroxidase-conjugated secondary antibodies were purchased from Santa Cruz Biotechnology (Dallas, TX, USA).

### Cell growth inhibition assay

EM23-induced growth inhibition was measured by MTT assay. CaSki and SiHa cells in log-phase growth were seeded in 96-well plates (6 × 10^3^ cells per well) and allowed to attach for about 12 h. Cells were then treated with a range of EM23 concentrations. The medium was removed 24 h later, and 50 μL of the same medium containing 5 mg/mL MTT was added to each well, and incubated for another 4 h. The medium was removed again, and 100 μL DMSO was added to each well and incubated for 15 min. Cell viability was determined by the absorbance at 570 nm as measured by microplate reader (Bio-Rad; Hercules, CA, USA), and the IC_50_ values of EM23 for each cell line were calculated using Origin 8 software (OriginLab, Northampton, MA USA).

### Flow cytometry analysis of cell cycle arrest and apoptosis

CaSki (3.0 × 10^5^ cells/mL) and SiHa (4.0 × 10^5^ cells/mL) cells were seeded in 6-well Petri dishes the day prior to the experiment. Cells were treated with 5, 15, or 20 μM EM23 for 24 h, then harvested, washed twice with ice-cold PBS, and fixed in 70% ethanol at −20°C overnight. Fixed cells were washed once with ice-cold PBS and re-suspended in 1 mL of staining reagent containing 100 mg/mL RNase and 50 mg/mL PI for 25 min in the dark. To assess apoptotic ratio, harvested cells were stained with Annexin-V-FITC/PI (KeyGEN; Nanjing, China) according to the manufacturer's instructions. Cell cycle arrest and apoptosis were analyzed by flow cytometry (BD FACSCalibur; Franklin Lakes, CA, USA). Fluorescence of PI and Annexin-V-FITC was monitored at 630 nm and 525 nm, respectively.

### Evaluation of mitochondrial membrane potential (MMP)

CaSki (3.0 × 10^5^ cells/mL) and SiHa (4.0 × 10^5^ cells/mL) cells were seeded in 6-well Petri dishes the day prior to the experiment. After treatment with 5, 15, or 20 μM EM23 for 24 h, cells were harvested, washed twice with ice-cold PBS, and incubated with JC-1 (10 μg/mL) in the dark for 15 min at 37°C. Cells were then washed three times with ice-cold PBS and analyzed by flow cytometry using emission wavelengths of 590 nm and 525 nm.

### Immunoblotting

After treatment with 5, 15, or 20 μM EM23 for 24 h, cells were harvested and washed twice with ice-cold PBS. For immunoblotting analysis, cells were lysed in RIPA buffer (Beyotime; Shanghai, China) for 30 min on ice, and then centrifuged at 12,000 g for 15 min. Supernatants were collected and protein was denatured at 100°C for 5 min. Equal amounts of protein were then separated on SDS-PAGE gels and transferred to PVDF membranes. Membranes were blocked with 5% nonfat milk at room temperature for 1 h, incubated with primary antibodies overnight at 4°C, and then with a horseradish peroxidase-conjugated secondary antibody at room temperature for 1 h. Protein bands were visualized by enhanced chemiluminescence (Millipore).

### Immunoprecipitation

For immunoprecipitation, equal amounts of protein from lysates prepared as above were co-incubated with Trx primary antibody and protein A magnetic beads (Santa Cruz) at 4°C overnight. The pellet was washed five times with ice-cold NP40 (Beyotime), resuspended in SDS-loading buffer (Beyotime), and analyzed by immunoblotting with Trx and ASK1 antibodies, respectively.

### Determination of ROS production

CaSki (3.0 × 10^5^ cells/mL) and SiHa (4.0×10^5^ cells/mL) cells were seeded in 6-well Petri dishes. After the treatment with 15 μM EM23 for 1–4 h, cells were incubated with 10 μM DCFH-DA for 15 min in the dark, washed three times with ice-cold PBS, and DCFH-DA fluorescence was measured at 525 nm by flow cytometry on a BD FACSCalibur.

### Quantitative real-time PCR

CaSki (3.0 × 10^5^ cells/mL) and SiHa (4.0 × 10^5^ cells/mL) cells were seeded into 6-well plates. Cells were harvested following treatment with 5, 15, and 20 μM EM23 for 24 h, and total RNA was extracted using Pure Link™ Viral RNA/DNA Kits (Invitrogen). The quantitative PCR of Trx and TrxR mRNA was performed using SYBR Green Real-time PCR Master Mix (Toyobo) as described by the manufacturer. Gene-specific primer pairs used in this study were as follows: TrxR sense 5′-TGTTGAATGAACAACTGTGC-3′ and TrxR antisense 5′-TCCTCAGCCAGTACATTGAC-3′, Trx sense 5′-CATAACCAGCCATTGGCTATT-3′ and Trx antisense 5′-GCATAATGTTTATTGTCACG-3′, GAPAH sense 5′-CACCCAGAAGACTGTGGATGG-3′ and GAPDH antisense 5′-GTCTACATGGCAACTGTGAGG-3′.

### Inhibition of TrxR in cell-free systems

TrxR (50 nM) and NADPH (200 μM) were incubated in 100 mM potassium phosphate buffer (pH 7.4) supplemented with 2 mM EDTA at room temperature for 4–5 min. Various concentrations of EM23 were added to the mixture and incubated for another 5 min. The reaction was started and TrxR activity was monitored by the increase in absorbance at 412 nm in the initial 80s after the addition of 2 mM 5,5′-dithiobis (2-nitrobenzoic acid) acid (DTNB), where TrxR mediated the reduction of DTNB into two molecules of 2-nitro-5-thiobenzoate anion (TNB) in the presence of NADPH. TrxR activity was expressed as percentage of control. For time-dependent incubation, TrxR was incubated with 10 μM EM23 in the above assay mixture for various time periods. TrxR activity was measured by DTNB reduction assay and was expressed as a percentage of the corresponding controls in the absence of EM23.

### BIAM-labeling assay

TrxR was incubated with EM23 in 20 mM Tris-HCl, 1 mM EDTA pH 7.5, and 200 μM NADPH at room temperature for 1 h, and then subjected to BIAM alkylation in buffers containing 100 mM Tris-HCl and 1 mM EDTA at pH 6.5 or 8.5 for 30 min. The protein samples were then denatured in SDS sample buffer (CST). BIAM-labeled TrxR was separated by SDS–PAGE, and detected with horseradish peroxidase (HRP)-conjugated streptavidin (Beyotime) and enhanced chemiluminescence (Millipore).

### Mass spectrometry analysis

Ac-Gly-[Cys-Sec]-Gly-NH_2_ tetrapeptide, mimicking the TrxR C-terminal redox-active site, was reduced by DTT prior to its addition to a reaction with 1.4 equivalents of EM23 for 30 min at room temperature. The reaction products were analyzed with an Agilent 6210 TOF LC/MS system (Agilent, Santa Clara, CA, USA) in positive ion mode.

### Molecular docking studies

Docking studies were performed using the molecular modeling software package SYBYL 8.0 (Tripos, USA). The ligand EM23 was charged with Gasteiger-Hückel and minimized by using the Powell's method with standard Tripos force field with a 0.005 kcal/(mol*Å) gradient. The maximum number of iterations during minimization was 1000. The minimum-energy structure was used for the subsequent docking calculations. The initial 3D structure of TrxR used for docking studies was retrieved from the Protein Data Bank (PDB) with accession code 2ZZB. The Surflex-Dock program implanted in SYBYL was used for the docking calculations. To generate the Surflex-Dock control file, the crystallographic ligand was extracted from the binding site of TrxR, and the residues within a 5.0 Å radius around the enzyme were defined as the active site. MOLCAD surface displayed in Fast Connolly pattern was generated for visualizing the binding mode of the docked protein-ligand complexes.

### Transfection with small interfering RNA (siRNA)

Trx and TrxR siRNA were purchased from Santa Cruz. CaSki and SiHa cells were seeded at 2 × 10^5^ per well in 2 mL antibiotic-free DMEM medium supplemented with FBS. Cells were transfected with 0.4 μg siRNA using Lipofectamine 2000 (Invitrogen; Carlesbad, CA, USA) per the manufacturer's protocols. After transfection for 24 h, cells were incubated with 15 μM EM23 for 24 h, and then collected for analysis by flow cytometry and western blot.

### Transmission electron microscopy

Cells treated with 15 μM EM23 for 24 h were harvested, washed twice with ice-cold PBS, resuspended in glutaraldehyde, and incubated for 20 min at room temperature. Ultrathin slices were prepared and examined under a Hitachi 7000 transmission electron microscope (Tokyo, Japan).

### Statistical analysis

All data are reported as the mean ± SD of three independent experiments performed in triplicate. The differences between two treatment groups were analyzed by two-tailed unpaired Student's *t* test; three or more groups were compared by one-way ANOVA multiple comparisons. *P*-values of < 0.05 were regarded as statistically significant.

## SUPPLEMENTARY MATERIALS FIGURES


