# Supplementary material for: EM23, a natural sesquiterpene lactone, targets thioredoxin reductase to activate JNK and cell death pathways in human cervical cancer cells
Source: Oncotarget. 2016 Jan 7;7(6):6790–808. doi: 10.18632/oncotarget.6828 (PMC4872749; doi:10.18632/oncotarget.6828)
Supplement: Supplementary file 1 [file oncotarget-07-6790-s001.pdf]

EM23, a natural sesquiterpene lactone, targets thioredoxin reductase to activate JNK and cell death pathways in human cervical cancer cells

Supplementary Materials

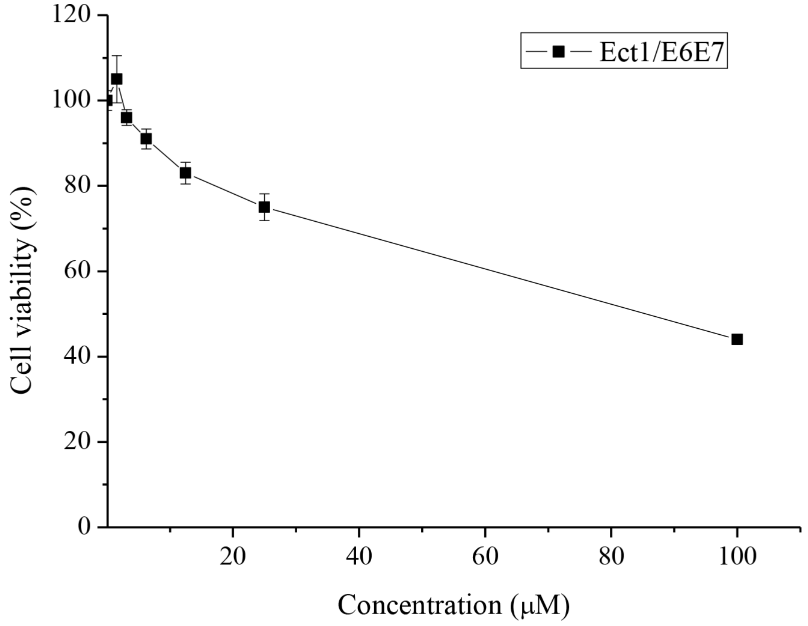

Supplementary Figure S1: Growth inhibitory effect of EM23 on human cervical epithelial Ect1/E6E7 cells. The effect of EM23 on the cellular growth inhibition of Ect1/E6E7 cells (ATCC, USA) was determined by MTT assay. Results demonstrated that EM23 showed moderate growth inhibitory activity against Ect1/E6E7 cells with IC50 value of 85.5 μM after 24 h treatment.

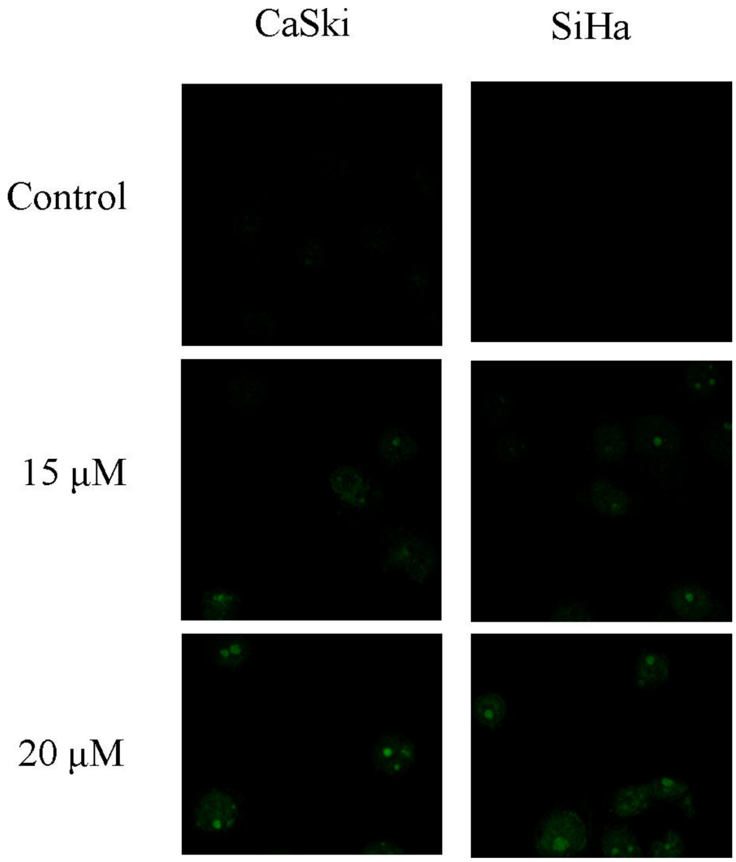

Supplementary Figure S2: Terminal deoxynucleotidyl transferase (TdT)-mediated dUTP nick-end-labeling (TUNEL) assay. DNA strand breaks in CaSki and SiHa cells were analyzed using the TUNEL assay, which is one of the most reliable methods for observation of apoptotic DNA cleavage. The technique was conducted as described by the manufacturer. Briefly, CaSki and SiHa cells were treated with 0, 15, and 20 μM EM23 for 24 h. The cells were fixed with 4% paraformaldehyde in PBS for 20 min at 4°C and immersed in 0.2% Triton X-100 for 5 min, then washed with PBS twice. The cells were preincubated for 5 min at room temperature in equilibration buffer and then incubated with 50 μl TdT incubation buffer at 37°C for 1 h in the dark. The reaction was halted by adding 2× SSC buffer for 15 min at room temperature. Samples were washed three times with PBS and then analyzed under a laser scanning confocal microscope. The experiment was repeated three times under each condition to ensure reproducibility. The images showed that only EM23-treated cells were TUNEL-positive as characterized by green fluorescence, which demonstrated the occurrence of DNA fragmentation.
